# Therapeutic Outcomes of ^177^Lu-PSMA Targeted Therapy in Patients with Metastatic Castration-Resistant Prostate Cancer: A Single-Center Study

**DOI:** 10.22038/AOJNMB.2022.64964.1454

**Published:** 2023

**Authors:** Seyed Ali Mirshahvalad, Saeed Farzanefar, Mehrshad Abbasi

**Affiliations:** 1 Research Center for Nuclear Medicine, Shariati Hospital, Tehran University of Medical Sciences, Tehran, Iran; 2 Department of Nuclear Medicine, Vali-Asr Hospital, Tehran University of Medical Sciences, Tehran, Iran

**Keywords:** Lutetium-177, Prostate Cancer, PSMA, Theranostic

## Abstract

**Objective(s):**

This study aimed to evaluate the therapeutic outcomes of ^177^Lutetium (^177^Lu)-PSMA-617 in metastatic castrate-resistant prostate cancer (mCRPC) patients, based on post-treatment imaging findings.

**Methods:**

All post-therapeutic scans were collected retrospectively from patients treated with 100-200 mCi ^177^Lu-PSMA-617 from March 2018 to December 2020 for mCRPC. Two independent readers interpreted the scans and visually categorized them into three strata: responsive, stable, and progressive. The responses were defined based on changes in the number of detected lesions, as well as the intensity of the hottest lesion. Data were registered, and the trend of changes was descriptively discussed.

**Results:**

Out of 36 patients (aged 67±8.8 years), 23 underwent at least two treatment cycles. Nineteen patients (82.6%) had bone metastases, 12 (52.2%) had nodal metastases, 5 (21.7%) had liver metastases, and 3 (13.0%) had lung metastases. Eleven patients (47.8%) were considered responsive in the post-therapeutic scans, two of which experienced complete eradication of the metastatic sites. Three patients (13%) were categorized as progressive, and 9 (39.1%) patients remained stable. Regarding mortality, nine patients died during the late follow-up (median of 24 months). In the surviving population, 65% reported no or mild pain in the final follow-up, based on a 5-point scale pain assessment.

**Conclusion:**

The treatment of mCRPC patients with ^177^Lu-PSMA-617 may limit their disease progression and preserve their physical performance, which are important factors in their survival and quality of life.

## Introduction

 Prostate carcinoma is a significant burden on every healthcare system as the second most common men-related cancer and the third rank in cancer mortality (1). Radio-ligand therapy (RLT), which has evolved to a great degree since 1898, recently showed promising results in treating prostate cancer patients (2). Tumoral lesions can be targeted efficiently by using prostate-specific membrane antigen (PSMA) as the ligand. The PSMA is a transmembrane protein serving as a surface marker present in many active prostate metastatic lesions. Although not prostate-specific, PSMA is over-expressed in a prostate cancer cell up to 1,000 times higher than in a normal tissue, which correlates with the Gleason score, distant metastasis, and hormone resistance (3). 

 The PSMA labeling has been performed using various radionuclides, showing the highest yield with ^90^Yittrium and ^177^Lutetium (^177^Lu). Further trials revealed that binding with the medium-energy beta-emitter ^177^Lu results in fewer adverse effects, especially concerning hematotoxicity. In addition, due to the short beta range (tissue penetration of <2mm) of ^177^Lu, less irradiation to the surrounding normal tissues can be achieved in small tumors (4). Moreover, the emitting gamma rays of ^177^Lu allow gamma imaging and gathering of information pertaining to the localization and dosimetry of tumoral lesions.

 The outcomes of ^177^Lu-PSMA targeted therapy have demonstrated an acceptable response rate in the metastatic castration-resistant prostate cancer (mCRPC) (5). It has also been documented that RLT decreases prostate-specific antigen (PSA) levels and ^177^Lu-PSMA therapy increases the quality of life and progression-free survival (6). The reported ample evidence regarding the efficacy of treating mCRPC patients with ^177^Lu-PSMA resulted in its recent Food and Drug Administration (FDA) approval, which can be a milestone in the targeted therapy (7). In the last decade, there were very few studies regarding ^177^Lu-PSMA in Iran, which showed similar results to the current literature in terms of the safety and efficacy of this therapy (8-10).

 In measuring efficacy, the response assessment of ^177^Lu-PSMA therapy is primarily based on the results of PSMA (labeled with both 68Gallium and 18Fluorine) positron emission tomography/computed tomography (PET/CT) and tracking patients’ serum PSA levels (11). The PSMA PET/CT is a diagnostic modality of choice to evaluate the pre-treatment status of patients regarding the extent of disease and expression of PSMA, as well as their interim and late response to the therapy. Although being a well-established modality with standardized semi-quantitative parameters, PSMA PET/CT is expensive at least for some patients. In addition to the proposed modalities in the follow-up, it is recommended that all patients undergo a post-treatment scan after each cycle (6, 11). This scan is interpreted for assessing the proper ligand targeting after receiving ^177^Lu-PSMA. However, its value over time and in the response assessment is unclear.

 This study aimed to report the outcomes of ^177^Lu-PSMA-617 therapy in mCRPC patients’ tumor burden (number and PSMA expression) in the center under study. The response assessment was based on the proposed criteria considering only post-treatment imaging findings.

## Methods

 In this retrospective study, data included all post-treatment scans of mCRPC patients treated with ^177^Lu-PSMA from March 2018 to December 2020. Patients with the following criteria were included in the study: 1) pathologically confirmed adenocarcinoma of the prostate, 2) diagnosis of mCRPC after anti-androgen treatment failure, 3) an Eastern Cooperative Oncology Group performance score of ≤2 with a life expectancy greater than six months, and 4) ^177^Lu-PSMA therapy and the post-treatment scan afterward. All patients had laboratory tests before admission to be excluded in case of bone marrow and urinary system or liver impairment. The ^177^Lu-PSMA-617 (will be called ^177^Lu-PSMA afterward) was prepared based on the Iranian society of nuclear medicine standards and the manufacturer’s instructions (Pars Isotope Co, Iran). The patients received 3,700-7,400 MBq (100-200 mCi) ^177^Lu-PSMA in each cycle. The dose adjustment was based on the patients’ weight, calculated as 74 MBq/kg (2 mCi/kg) with the mentioned minimum and maximum doses. Patients underwent various cycles based on their eligibility with an expected four standard cycles (further cycles were decided after evaluating the treatment efficacy) and an interval of 6-8 weeks.

 All included patients were treated in the ward and presented for the post-treatment scan 24 to 48 h after the infusion of ^177^Lu-PSMA. For treatment, 25 cc/kg±10% dextrose water (5%) was infused before and after the administration of ^177^Lu-PSMA. Patients were observed for 4 h and discharged after the third urination and radiation at a one-meter distance of less than 25 µSiv/h. Whole-body imaging was performed using a dual-head gamma camera (Anyscan, Mediso, Budapest, Hungry) with a photopeak of 113, speed of 12 cm/min, and matrix of 256×1024. If needed, an additional pelvic single-photon emission computed tomography (SPECT) was performed.

 The imaging of each patient was interpreted by two independent readers blinded to the results of the previous or following cycle(s). After this process, the cycle reports of each patient were ordered based on the date of imaging, and then, by reviewing the trend of changes, patients were categorized into three strata, as described in Table 1. In case of discrepancies in each step, a consensus was made. Data were registered and the trend of changes in the post-therapy scans was descriptively discussed.

## Results

**Figure 1:**
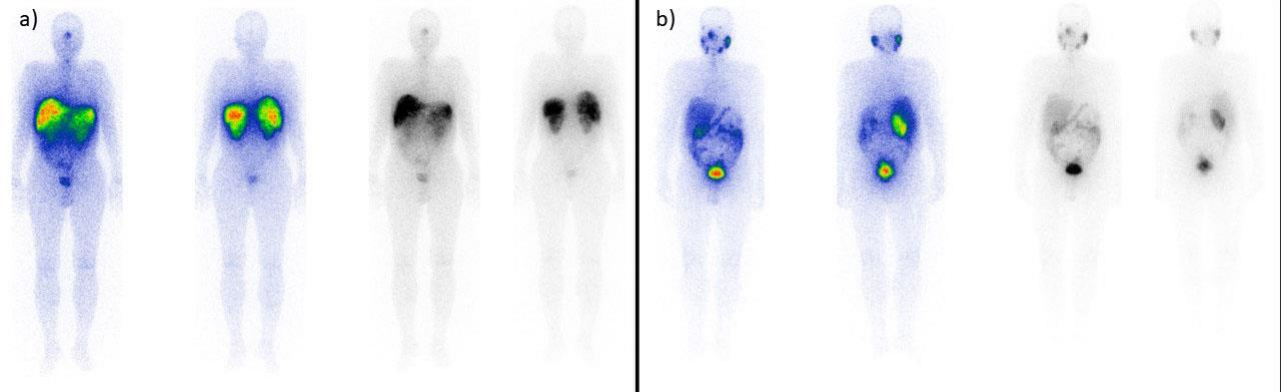
A 55-year-old male patient with documented liver metastases was referred to the center under study for therapy. On the planar images (**a**), extensive liver metastases are depicted. This patient underwent three treatment cycles and was categorized as a responsive patient resulting in the eradication of the noted lesions (**b**)

**Figure 2:**
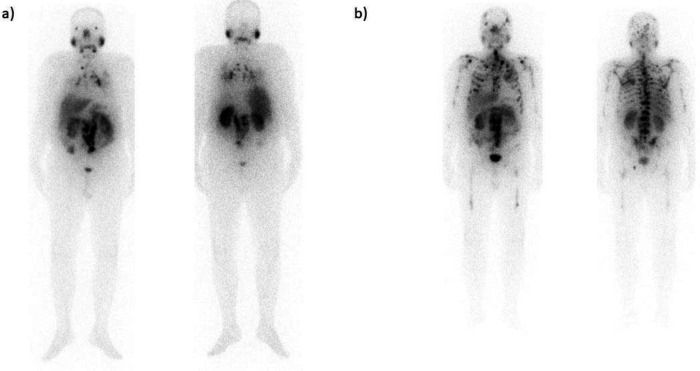
A 63-year-old male patient with metastatic prostate cancer. Pulmonary metastases are evident, accompanied by skeletal and nodal metastases (**a**). The patient underwent four cycles of therapy, and the therapeutic cycles were terminated due to the disease progression (**b**)

 Out of 36 patients aged 67±8.8 years, 23 underwent at least two treatment cycles. Eight patients received four cycles or more, and 15 patients received two or three cycles. Out of 23, 19 (82.6%) had bone metastasis, including 7 (30.4%) patients with super-scan and 7 (30.4%) with pelvic bones involvements, and 12 (52.2%) had nodal metastasis, including 7 (30.4%) pelvic lymphadenopathies and 13 (56.5%) abdominal, mediastinal, cervical, or axillary lymphadenopathies. Moreover, 5 (21.7%) patients had liver metastasis, and 3 (13.0%) had lung metastasis (Figures 1 and 2). 

 Noteworthy, 13 cases who received only one treatment cycle were those who had not come to the center for the later cycles. The reasons included no avid lesions in the first post-treatment scan, the limited disease after the first cycle considering the treatment’s cost-effectiveness, under-controlled disease based on the opinion of a multidisciplinary team, and patient refusal. None of the patients’ therapy was terminated due to the adverse effects.

**Figure 3:**
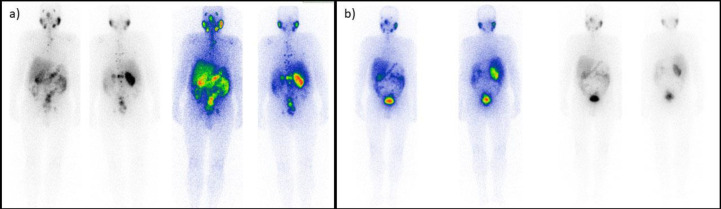
Responsive patient. A 62-year-old male patient with metastatic castrate-resistant prostate cancer underwent five cycles of treatment. The initial scan (**a**) shows bone and lymph node avid metastases, all eradicated after the last cycle (**b**)

**Figure 4:**
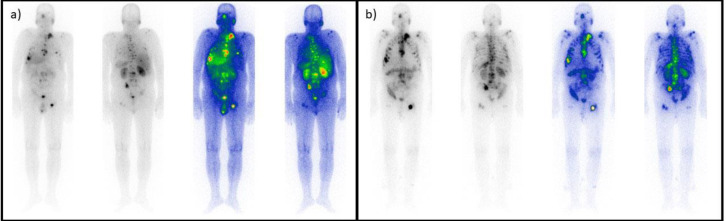
Progressive patient. A 72-year-old male patient presented with several skeletal and nodal metastases (**a**). After the second (last) cycle (**b**), the scan indicated progression in the tumoral burden, and the treatment was terminated

 Eleven patients (47.8%) were considered responsive in the post-therapeutic scans by ^177^Lu-PSMA, based on the proposed criteria, two of which experienced complete eradication of the metastatic sites (Figure 3). Three patients (13%) were categorized as progressive (Figure 4), and 9 (39.1%) patients had stable disease in their scans.

 In the follow-up (median=24 months) of all 36 patients, 9 patients died, including 1 progressive patient (cancer-related, age=71 at the initiation of therapy), 4 stable patients (mean age=76 at the initiation of therapy), and 4 responsive patients (mean age=75 at the initiation of therapy). Among the stable patients, three of them deceased following non-related (cardiovascular) events, and one was considered cancer-related. Among the responsive patients, two died of coronary artery disease. The therapy of the two other patients was terminated due to the decision of their referring physician (or the lack of patient’s treatment adherence), which more likely seemed to be based on the belief that the disease was under control and there was no need for continuing the therapy. After the termination of the therapy (after two cycles), they experienced a deterioration in their disease.

 In the surviving patient population, at the time of the final follow-up on 26 May 2021, based on a 5-point scale assessment of the pain severity (1=no pain; 5=severe pain), 2 (14%) patients reported a score of 4, 3 (21%) patients scored 3, 4 (29%) patients scored 2, and others (36%) scored 1. Overall, 11 (79%) patients mentioned that they experienced pain relief after receiving ^177^Lu-PSMA therapy, compared to their basal pain scores.

## Discussion

 This study reported a single-center experience of ^177^Lu-PSMA therapy in mCRPC patients. The results showed that the majority of patients responded to ^177^Lu-PSMA therapy or at least remained stable in the later cycles. This finding was valuable since patients were all progressive on their previous treatments (castration-resistant). In terms of their physical performance, living patients experienced acceptable pain relief in an approximately two-year follow-up. However, a mortality rate of 25% was documented.

 The PSMA is a ligand to a transmembrane hydrolase enzyme, which can be labeled with ^68^Gallium and ^99m^Technetium for imaging purposes by PET and SPECT, respectively. Furthermore, it can be labeled with ^177^Lu and Act-225 for therapeutic purposes (12-14). The ^177^Lu is a beta emitter with a half-life of 160 h and a preferential urinary excretion. The radiation of ^177^Lu-PSMA to non-metastatic tissues is low, and radiation to the medical staff, caregivers, as well as the public, is negligible approximately 4 h after the infusion of the radiopharmaceutical and the urinary excretion (15, 16). The ^177^Lu-PSMA attaches to the PSMA peptide in the membrane and then internalizes to the cell. Beta radiation travels about 2 mm in the tissue and destroys the PSMA presenting cells, mainly the metastases from prostate cancer (4). Many non-prostatic cells express PSMA, including lymphocytes and hepatocytes. However, the clinical significance of irradiating bone marrow and the liver is not a concern considering the evidence suggesting the low prevalence of the related side effects (such as hepatotoxicity, nephrotoxicity, and high-grade bone marrow suppression)(6,11,17).

 Since the introduction of ^177^Lu-PSMA, several studies have been published regarding the efficacy of this therapy in mCRPC patients (6, 18-20). Recently, the findings of the VISION study (21) provided high-quality evidence for this treatment, and the FDA approved ^177^Lu-PSMA therapy for progressive mCRPC patients who are eligible candidates. In addition to the reported high responses and improvements in progression-free and overall survival, studies have found that this treatment can increase the mCRPC patients’ quality of life and result in pain relief.

 The key difference between the current study and the available literature was the manner of evaluating response to the treatment. Although in the proposed guidelines, the standard approach for assessing response is based on measuring PSMA PET/CT findings and its semi-quantitative parameters (22-24), the authors adopted qualitative criteria for evaluating post-treatment scans and tracking the response to ^177^Lu-PSMA therapy. Having said that, the authors considered the lesion number and a 5-point scaling with the hepatic background reference to be more objective in decision-making. This assessment was based on considerations regarding the cost and availability of PSMA PET/CT since some patients refuse to follow the treatment or at least undergo the recommended PSMA PET/CT after every two cycles because of the expenses. Moreover, it is believed that in addition to the evaluation of ligand targeting and the avidity of lesions on post-treatment scans, there might be an advantage of the post-treatment scans in terms of patients’ response trends.

 In this study, two patients experienced a complete response to the treatment with the eradication of all metastatic sites, and roughly 48% of patients illustrated improvement in the lesions. Another 39% of patients in the current sample had stable disease while they had experienced failure with previous therapies before initiating the systemic targeted therapy with ^177^Lu-PSMA. Furthermore, more than two-thirds of the included patients claimed they were experiencing minimal or no pain, resulting in an acceptable performance status provided by ^177^Lu-PSMA.

 The overall survival of mCRPC patients should have been about 11 months without RLT (25). The ^177^Lu-PSMA therapy seemed to prolong the survival of patients so that only nine mortalities were reported during the 26±7 months of follow-up (25% mortality in about two years) in the current study. However, especially since 13 patients received only one cycle, a larger population with standardized cycles is needed to reach such a fact.

 It is worth mentioning that the present study was seriously limited by the lack of clinical data, especially the PSA levels. It was also impossible to gather other imaging results, particularly PSMA PET/CT, of patients to investigate their disease status further. These remarkable limitations were due to the lack of medical records in the center under study, which was not possible to tackle. Nevertheless, the results of the current study may provide a deeper insight for nuclear physicians with only limited access to post-therapy images, compared to reports from their urologist or oncologist counterparts. Larger studies with detailed data are needed for a comprehensive evaluation of the post-therapy imaging to assess the efficacy of ^177^Lu-PSMA therapy and optimize an individualized treatment for prostate cancer patients.

## Conclusion

 This limited experience in our center showed that treating mCRPC patients with ^177^Lu-PSMA may limit their disease progression and preserve their performance, which are important factors in their survival and quality of life.

## Ethics approval and consent to participate

 All human studies have been approved by the Tehran University of Medical Sciences Ethics Committee and therefore, have been performed following the ethical standards laid down in the 1964 Declaration of Helsinki and all subsequent revisions.

## Conflict of interest

 The authors declare that they have no conflict of interest.
